# On the Technology Acceptance Behavior of Romanian Preschool Teachers

**DOI:** 10.3390/bs13020133

**Published:** 2023-02-05

**Authors:** Dana Rad, Anca Egerău, Alina Roman, Tiberiu Dughi, Gabriela Kelemen, Evelina Balaș, Adela Redeș, Maria-Doina Schipor, Otilia Clipa, Liliana Mâță, Roxana Maier, Gavril Rad, Remus Runcan, Csaba Kiss

**Affiliations:** 1 Center of Research Development and Innovation in Psychology, Faculty of Educational Sciences Psychology and Social Sciences, Aurel Vlaicu University of Arad, 310130 Arad, Romania; 2 Academia Oamenilor de Știință din România, 50085 Bucharest, Romania; 3 Faculty of Science of Education, Ștefan cel Mare University of Suceava, 720229 Suceava, Romania; 4 Teacher Training Department, Vasile Alecsandri University of Bacau, 600115 Bacau, Romania; 5 Faculty of Psychology and Educational Sciences, Hyperion University of Bucharest, 030615 Bucharest, Romania

**Keywords:** technology acceptance behavior, sequential mediation analysis, preschool teachers, perceived enjoyment, compatibility, intention to use and actual use of technology

## Abstract

This study investigates how compatibility and perceived enjoyment affect the link between intention to use and actual technology use in Romanian preschool education, building on earlier studies. Methods: 300 participants were invited to participate in this research from 15 Romanian counties. 182 preschool teachers’ questionnaires were utilized for data analysis after the return and screening of responses. A valid and accurate scale evaluating preschool teachers’ behavior towards technology adoption was included in the questionnaire, along with self-reported demographic data, professional identification, and other information. Data was analyzed using SPSS V.16. Results: (1) Intention to use, compatibility, perceived enjoyment, and actual use were positively associated. (2) The effect of compatibility and perceived enjoyment on the link between intention to use and actual technology use was carried out in the following way: Intention to use → Compatibility with technology → Perceived enjoyment → Actual use. We hypothesize that intention to use affects compatibility, compatibility affects perceived enjoyment, and, lastly, perceived enjoyment affects actual use. For a more robust validation of results, we have also modelled this relationship with the Radial Basis Function (RBF) neural network. Conclusion: Compatibility and perceived enjoyment partially mediate the relationship between intention to use and actual technology use in class by Romanian preschool teachers. According to the theory of planned behavior, this study brought to light the intricacy of the relationship between preschool teachers’ intention to utilize technology in the classroom and their actual usage of it. Limitations and implications are discussed.

## 1. Introduction

Modern classrooms, including those for early childhood education, whether they are face-to-face or online, rely heavily on technology. When implemented properly, educational technology can improve children’s learning outcomes. However, it might be difficult to choose the right instructional technology and successfully integrate them. It involves cooperation between all parties, intentions, and technological knowledge.

The goal of technology acceptance in early childhood education is to enhance the learning and developmental outcomes for the child. Utilizing technology for its own sake is not appropriate. Instead, it serves as a tool to aid in the accomplishment of educational goals. When choosing and using technology in the classroom, educators must take a variety of variables and concerns into account. What educational and developmental goals can this technology, for instance, aid a youngster in achieving? How will it improve a child’s desire to learn? Is the technology suitable for a child’s developmental stages and specific learning requirements?

Multimedia technology may be used to engage learners with various learning requirements through instructional and assessment activities. These technologies allow for the flexible distribution of material, enabling the differentiation of courses and activities in accordance with unique learning preferences. All children may have easier access to legitimate learning opportunities as a result. The value of interaction and its relationship to active learning must be emphasized. Young children may only absorb rote facts when they consume technological material passively without actively considering and reflecting on it. On the other hand, active technology use may motivate youngsters to study. Active technology depends on the user’s deliberate interaction and engagement with the material. The dynamic use of educational technology is encouraged by interaction and co-viewing with adults and peers, and reflection boosts their beneficial effects. For young learners, interactive involvement is essential.

Despite more computer availability with modern smart applications, technology is still seldom used in the classroom, particularly in early childhood education. There have been studies exploring how teachers’ attitudes and beliefs about technology affect infrequent use [1,2,3], but there have been relatively few large-scale quantitative studies focusing on preschool teachers, a crucial group given the ongoing discussion about the role of technology in children’s lives.

The behavior of technology acceptance in educators, in general, and in preschool teachers, in particular, have drawn the attention of education researchers in the last two decades; however, most of them have used information technology acceptance theories such as, according to [4] and [5], the Theory of Reasoned Action (TRA) [6], the Theory of Planned Behavior (TPB) [6], the Social Cognitive Theory (SCT), the Technology Acceptance Model (TAM) [7], the Technology, Organization, and Environment framework (TOE), the Model of PC Utilization (MPCU) [8], the Motivational Model (MM) [7], the Combined-TAM-TPB Model [8], and the Innovation Diffusion Theory (IDT) [9].

The (TAM), created by [7], sheds light on the forces that shape acceptance tendencies as well as the likelihood that a given technology would be accepted. In this model, the user’s intention to use the technology, which serves as the primary predictor of its actual usage, is largely influenced by how easy it is to use and how helpful it is regarded to be.

Technology acceptance by educators has been investigated in pre-service teachers [10,11,12,13]; preschool teachers [14,15,16,17,18,19]; school teachers [4,20,21,22,23,24,25,26,27]; school counsellors [28]; and academics [29,30,31,32]. In parallel, overuse of technology and its consequences on children, adolescents, young adults, and adults’ welfare have also been scrutinized by researchers [33,34,35].

The research of preschool instructors’ attitudes towards technology is still in its full development. The authors of [14] used the Unified Theory of Acceptance and Use of Technology (UTAUT) model (in which [6] had incorporated the most reliable TAM constructs—perceived usefulness of the technology and perceived ease of use) improved by [36] and the Use of Technology model to examine the first-time acceptance of socially assistive robots (SAR) by preschool and primary school teachers. The authors of [15] used the Technological Pedagogical and Content Knowledge (TPACK) theory, which is an expansion of the concept of Pedagogical and Content Knowledge (PCK), to examine preschool teachers’ beliefs and practices in the integration of technology into class to engage young children in learning sciences. The authors of [17,37] carried out a cross-sectional study to better understand how preschool instructors use digital technology (DT) in early childhood education. However, the availability of DT in early childhood classrooms and the use of these tools by preschool instructors received the majority of their focus. In research, [18] looked at pre-service preschool teachers’ adoption of Educational Robotics (ER) and their self-efficacy towards ER. They identified a substantial difference in the acceptance of ER in the areas of reported ease of use, enjoyment, attitudes, and self-efficacy, between the beginning and the completion of the ER training. The authors of [18] looked through three databases for publications on Educational Robotics (ER) (preschool) teacher training, discussed the landscape of ER teacher training research, and offered suggestions for organizations planning to implement ER teacher training programs.

Only [16] used an adjusted model to analyze the determinants of preschool teachers’ embrace of technology. The results of this investigation, which was carried out in the context of the COVID-19 pandemic, revealed that: intention to use was moderate to high; perceived usefulness was a direct significant predictor of intention to use; perceived ease of use was a direct significant predictor of intention to use; perceived usefulness was affected by perceived ease of use; perceived usefulness was affected by job relevance; and computer self-efficacy was a positive factor towards perceived ease.

The scarcity of studies carried out on technology acceptance in preschool education research was a determining factor in conducting this research.

### 1.1. Literature Review

Educational technology appears to be more and more integrated into early childhood education. In general, perceptions towards educational technology are favorable. Contrarily, beliefs are more likely to be based on the social argument that children need access to technology since it is a part of their everyday life than on the pedagogic argument that technology enhances learning. Reviewing materials could be needed to ensure that practices and policies are more centered on teaching and learning [38].

In order to better understand how new technologies could be influencing children’s literacy within academic settings, studies concluded that more in-depth exploratory research in this area is necessary that takes into account how digital techniques within academic settings relate to other aspects of children’s literacy development [39].

According to a recent study’s findings, the majority of instructors learn their computer knowledge and abilities via personal experience, and they believe that using computers in early childhood education is suitable. Additionally, it was said that most teachers use computers in their curricula 1–2 times each week and utilize them to enhance everyday tasks in daily schedules. Additionally, it appears that the instructors mostly utilize computers for music-related activities and that they want to employ computer-based activities to enhance children’s cognitive development [40].

Although educational research studies suggest that using technology may help students learn, its adoption is typically hampered by several constraints. Researchers have identified the general barriers that K-12 schools face when integrating technology into the curriculum for instructional purposes, which are as follows: (a) resources, (b) institution, (c) subject culture, (d) attitudes and beliefs, (e) knowledge and skills, and (f) assessment [41].

Research on preschool teachers’ impressions of technology acceptance has been chastised for failing to take into account the distinctive pedagogical qualities of early childhood education. A recent qualitative study investigated preservice teachers’ opinions of technology usage through the lenses of education, socialization, and care. The results indicate that a preservice teacher’s stance on technology use depends on the lens through which they see technological adoption. The most important elements influencing the variation in dynamics across and within the frameworks were the children’s ages and individuals’ perceptions about the children’s access to technology at home [42,43].

Key findings from a recent meta-analysis suggest that technology use had a favorable influence on 94% of the results reported in the ECEC research [44].

On the other hand, other research has indicated that early childhood instructors have limited access to computers and positive, yet reserved, opinions towards their use. Computer use at home, technological experience, and in-service training all appear to have a major impact on teachers’ attitudes [45].

In another study, training offered incentives for future ECEC professionals to change their favorable opinions about utilizing digital tools in the classroom. The findings demonstrated that the students’ instruction was a critical component in enhancing their perceptions of their own digital competence [46].

In another study, it was shown that ICTs have been utilized in the analyzed preschools in a variety of ways, such as a tool to improve existing practices, a cultural mediator, a way to amuse young children, and a way to communicate and record information. The paper also examines the perspectives held by educators who think that the use of ICT in early childhood education is inappropriate by examining their attitudes and beliefs on that topic [47,48].

The findings showed that preservice teachers had a favorable view of the role that technology plays in early childhood educational settings. Their level of satisfaction with their preparation for integrating technology into the classroom, however, was less favorable. The preservice staff members highlighted many knowledge gaps, including those that concerned how to involve children in digital and technical activities and how to integrate technology into practice. The study results suggest that future educators need to develop stronger curricula that addresses these concerns and children’s learning by integrating technology into their teaching practices with more assurance and competence to be able to assist teacher education programs [49].

Thus, most research conclusions showed that the majority of in-service teachers held positive opinions regarding how technology should be used in preschool instruction. Results also demonstrated the necessity of providing enough technology in early educational settings. Additionally, in-service preschool instructors highlighted that they anticipate receiving support from the preschool curriculum and a number of initiatives designed to improve their technological literacy. Finally, instructors were aware of both the potential benefits and drawbacks of technology.

Despite this good impression and attitude towards using ICT to facilitate playful learning and children’s development in young children outside the classroom [50,51,52], teachers are less likely to use ICT in teaching and play activities in their classrooms. The main perceived obstacles to the use of computers in preschool educational environments included a lack of finance, a lack of technical and administrative assistance, and a lack of training opportunities. The following four barrier-factors were identified: “class conditions”, “lack of support”, “lack of confidence”, and “lack of equipment”. Lack of assistance and classroom circumstances were directly and significantly impacted by teachers’ technological confidence. The more comfortable instructors are using technology, less assistance is required and less unfavorable classroom conditions are felt. Higher likelihood of computer use in class was caused by both teachers’ comfort with technology and the presence of a computer [53].

### 1.2. Current Research

Currently, a lot of research has focused on the relationship between intention to use, compatibility with technology, perceived enjoyment, and actual technology usage, but very little research has looked at their possible influencing processes. In order to investigate their possible influencing mechanisms, this study used the preschool teacher’s reported enjoyment and compatibility with technology as mediating factors, intention to use as an independent variable, and actual technology usage as a dependent variable. The problem of increasing the career flexibility of pre-service preschool teachers becomes vital given the pressing demand for their growth. In order to increase the career flexibility of preschool teachers, this research will examine the potential influencing processes of these factors.

Numerous studies [54,55,56,57,58,59] have shown that how users perceive the usability, utility, enjoyment, and service quality of technology will all affect people’s views towards using it. The self-efficacy theory and motivation theory both support the notion that people who are assured of their skills regard technology engagement as enjoyable and recognize that a task’s utility would function more effectively in circumstances where technology is used [58].

The intrinsic motivator of perceived enjoyment stresses the usage process and stands for the delight and satisfaction that come from utilizing a technology. The attitude towards utilizing a certain technology is positively connected with the experience of satisfaction. Having pleasure is one of the main reasons individuals use technology for online education [59]. Learners’ attitudes towards using new technologies will be more favorable if they can have fun while doing it [60]. It has long been recognized that attitude has a significant role in intention. Today’s preschool teachers are likely involved in online education and have developed opinions about it that range from favorable to unfavorable [16,60].

The TAM model states that intentions to utilize technology and actual use are influenced by perceived enjoyment and compatibility with technology.

**Figure 1:**
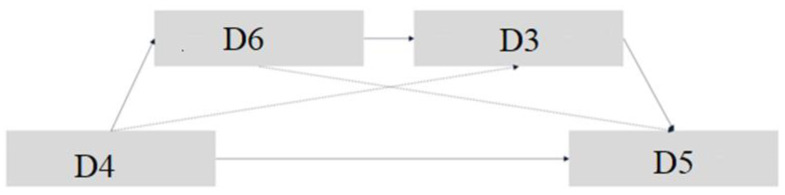
Hypothesized conceptual model. D4. Intention to use → D6. Compatibility → D3. Perceived enjoyment → D5. Actual use.

The theoretical hypothesis model is depicted in Figure 1 and we will further analyze the following hypothesis in light of past research:

Hypothesis: Between intention to use and actual use, compatibility and perceived enjoyment are complementary sequential mediators.

## 2. Materials and Methods

### 2.1. Research Design

This research employed a cross-sectional quantitative approach. A cross-sectional design allows for the simultaneous investigation of several variables, such as intention to use, compatibility with technology, perceived enjoyment, and actual use of technology by preschool teachers. A valid TAM-ECEC scale was used to collect responses for the research variables: intention to use, compatibility with technology, perceived enjoyment, and actual use of technology by preschool teachers.

We have further employed a correlational analysis to depict the relationship between variables. The next step was to test our hypothesis involving sequential mediating effects analyses, considering compatibility with technology and perceived enjoyment as complementary sequential mediators in the relationship between intention to use and actual technology use.

To further test the robustness of results, we have also modelled this relationship with a Radial Basis Function (RBF) neural network, a specific kind of Artificial Neural Network utilized for function approximation. RBF neural networks are three-layered models, they use universal approximation and train more quickly than other neural networks [61]. The RBF approach will use the same hypothesized conceptual model, with the dependent variable being D5. actual use and independent predictors being intention to use, compatibility with technology, and perceived enjoyment. This in-depth analysis will further reveal the predictive significance of intention to use, compatibility with technology, and perceived enjoyment over actual technology use in class, to further reveal the psychological mechanisms that play a role in between intention to use and actual technology use in preschool teachers.

### 2.2. Participants and Procedure

Data were collected from 15 school inspectorates from different western Romanian counties that were previously involved in a national training program for inclusion and qualitative preschool education financed by the Romanian Educational Ministry. Informed consent was acquired from all preschool teachers that agreed to take part in this research. Confidentiality and voluntary involvement were upheld as ethical norms. A total of 350 online questionnaire invitations were sent to previously training participating preschool teachers and 182 valid questionnaires were returned, with 100% valid responses. The data consisted of 182 female preschool teachers’ responses (100%). The age range of the teachers who answered our survey was 23 years old to 62 years old, with a mean of 42 years. The teachers’ cumulative experience in the field of preschool instruction ranged from 2 to 43 years, with a mean of 20 years. All of our responders have a degree in preschool and elementary education that has been granted government accreditation.

### 2.3. Measures

The participant’s age, gender, educational level, and city of residence were all reported on the personal information form.

Based on the theory of planned behavior, the TAM in early childhood education and care (TAM ECEC) is a scale that evaluates the technological acceptance of ECEC professionals in an online learning environment.

The preschool teacher is envisioned as an agent of change for ECEC’s embrace of technology, and they further suggest extending the conventional TAM while carefully taking into account ECEC-specific factors. This study employed the TAM scale to gather empirical information on Romanian preschool teachers’ technological literacy.

The online poll consists of 27 items, and respondents are requested to rate each one on a scale of 1 to 5 (where 1 stands for strong disagreement, and 5 stands for strong agreement). Reverse scoring is used for items 22, 23, 24, 25, and 27, thus, 1 becomes 5 and vice versa [42]. Higher scores showed more frequent usage of technology in the classroom. The validity and reliability of this measure among Romanian preschool instructors was satisfactory [42]. With the exception of D4 and D5, which are single elements and reflect the behavioral desire to use and actual usage of technology, respectively, Cronbach’s alpha for the dimensions D3, D6, and D7 in the current research sample was 0.91 and 0.91, respectively. The TAM ECEC online questionnaire had a computed Cronbach’s alpha of 0.93%, which indicated high reliability coefficients.

The average score on the scale was 24.77, with a variance of 48.68 and a standard deviation of 6.97, with actual usage M = 2.92, SD = 1.15, perceived enjoyment M= 3.25, SD = 1.03, intention to use M = 3.17, SD = 1.17, and compatibility M = 3.00, SD = 1.04. Additionally, both a Hotelling’s T-squared F of 44.29 at a *p*-value less than 0.01 and a Cochran’s alpha of 322.04 at a *p*-value under 0.01 were discovered. All of the above statistics were in favor of the TAM scale’s validity.

### 2.4. Data Analysis

Descriptive statistics were used to analyze the data along with correlation in SPSS 26.0, and Model 6 of Hayes was employed to evaluate the sequential mediating effects of compatibility and perceived enjoyment on the link between intention to use and actual technology usage [62]. Using the bias-corrected bootstrap method, the indirect influence was tested. The 95% confidence interval (CI), if the indirect influence was significant, did not include 0. To further test the robustness of results, we have also modelled this relationship with a Radial Basis Function (RBF) neural network, using the RBF facility in SPSS.

## 3. Results

### 3.1. Correlation Analyses

The correlation analysis findings suggest that perceived enjoyment was positively correlated with intention to use, compatibility, and actual use. Compatibility with technology was positively associated with intention to use and actual use. Perceived enjoyment was positively associated with compatibility, as depicted in Table 1.

### 3.2. The Sequential Mediating Effects Analyses

The correlation between the intention to actually use technology, compatibility with technology, and felt enjoyment of using technology in preschool instruction suggests additional investigation of the mediating role of compatibility with technology and perceived enjoyment [63].

Model 6 of Hayes was employed to evaluate the sequential mediating effects of compatibility with technology and perceived enjoyment on the connection between intention to use and actual usage. All variables were standardized.

In our sequential mediation, there is the independent variable: intention to use; the dependent variable: actual use; and two mediators: compatibility with technology and perceived enjoyment when using technology in preschool teaching. The two mediators do an increasingly better job at predicting the dependent variable.

The independent variable had a significant positive effect on the dependent variable (b = 0.53, t(180) = 6.82, *p* < 0.001). As theorized, this effect was sequentially mediated by the first mediator and the second mediator. The indirect pathway of the effect of the independent variable on the dependent variable via the two mediators was significant (b[indirect] = 0.77, t(180) = 17.57, *p* < 0.001). This pathway partially accounted for the overall impact of the independent variable on the dependent variable with the direct effect being still significant (b[direct] = 0.52, t(180) = 6.82, *p* < 0.001). The additional 22 percent in explaining the variance of actual technology use accounted for the introduction of the sequential mediators that clearly prove that compatibility with technology and perceived enjoyment of technology used by preschool teachers are crucial elements when trying to adopt more technologically accepting behaviors in preschool education.

The sequential mediation model explained an overall 66% of the actual technology use variance, which represented a very high percentage. This result is supported by the technology acceptance model theoretical framework and by practical results reported in the literature. Overall, this is the first mediation study that looks at compatibility with technology and perceived technology enjoyment as sequential mediators instead of single or parallel mediators.

### 3.3. RBF Neural Network Modelling of the Variable’s Relationship

**Figure 2:**
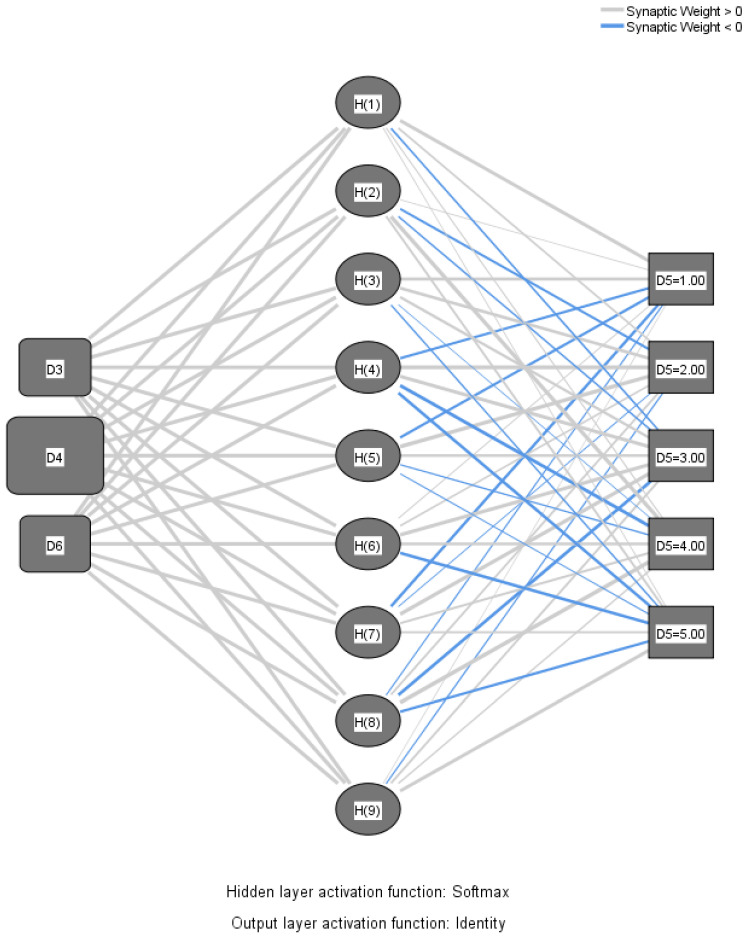
RBF architecture.

In order to employ the RBF neural network technique, we used the path Analyze/Neural Networks/Radial Basis Function in SPSS V.26 program. We proceeded with designing the architecture as follows. First, we selected the dependent variable as actual technology use, followed by the 3 parameters in the covariates section: intention to use, compatibility, and perceived enjoyment. We chose the standardization method for the rescaling of covariates. In the partition section, we chose to randomly assign cases based on relative number of cases: 70.3% training and 29.7% testing, with no partitioning variable to assign cases. Our RBF neural network was created with four main objectives in mind: to maximize performance, to reduce computational resources used during training, to increase the degree of automaticity by reducing the number of decisions that must be made by a human during the design process, and to reduce the complexity of the model, specifically the size of the network. As a result, we chose automatic architecture selection. The automated architecture design option suggested using the Softmax function for hidden layer activation and the Identity function for output layer activation. Figure 2 shows the FBF architecture with all the layers mentioned previously.

In the model summary, we discovered a cross entropy error of 35.586 and a training sample percentage of inaccurate predictions of 43%. A cross entropy error of 13.733 and a percentage of inaccurate predictions of 35.2% were found in the testing sample. Thus, 64.8% of the categorized data are appropriately allocated to the actual technology use value (dependent variable), which represents a considerable level of accurate classification rates in the testing sample, regarding the fact that the output variable was designed with five different data classification points (Likert’s 1 to 5 answers to single item D5. Actual use).

Additionally, Table 2 displays the proportion of correctly classified data points for each rating (1 to 5) in the training and testing samples. For example, with an overall accuracy percentage of 7.0%, data point 5 (very high actual use) had the lowest correct percentage in the training sample of 45.5%. With an overall accurate percentage of 11.1%, data point 5 (very high actual) in the testing sample had one of the highest correct classification rates of 71.4%.

When applying the RBF model, the area under the curve (AUC) value exhibited great overall performance. We received a value of 0.927 for data point 1 (very low actual use), a value of 0.814 for data point 2 (low actual use), a value of 0.798 for data point 3 (medium actual use), a value of 0.859 for data point 4 (high actual use), and a value of 0.952 for data point 5 (very high actual use). An AUC of 0.5 often indicates no discrimination, whereas values between 0.7 and 0.8 are regarded as good, between 0.8 and 0.9 as excellent, and values beyond 0.9 as remarkable.

Results from the RBF model’s training phase showed that intention to use, perceived enjoyment, and compatibility with technology were all significant variables for predicting actual use of technology in class by preschool teachers; their respective normalized importance were 100%, 61.3%, and 59.2%, respectively, and the results were consistent with other reported studies [64,65,66,67,68].

## 4. Discussion

This study used a sequential mediation approach to examine the relationship between preschool Romanian teachers’ desire to utilize technology in the classroom and their actual usage of it. The study’s findings revealed that the connection between preschool instructors’ desire to utilize technology and actual use was partially mediated by perceptions of enjoyment and compatibility with it. Our research also supported the connection between preschool instructors’ intention to utilize technology and their actual usage of it [59,60,61,62,63,64,65,66,67,68,69,70,71,72,73]. The results showed that intention to use affects actual use of technology through two sequential mediators: perceived enjoyment and compatibility with technology.

This will enable us to better understand the relationship between preschool teachers’ intentions to utilize technology and their actual use of it. It will also make it easier for educational managers to modify training programs to improve preschool teachers’ actual usage of technology.

In order to confirm the partial mediating function of perceived enjoyment on intention to use and actual technology usage, which was consistent to some extent with prior research, we first tested the relationship between perceived enjoyment and intention to use and actual technology use [74,75]. In more detail, we discovered that preschool instructors who intended to utilize technology more frequently also increased both their real and perceived levels of enjoyment. However, if the preschool instructors have a low level of intention to use, their perceived enjoyment levels will decrease and their actual usage of technology will also decrease, finally resulting in a lower level of career flexibility.

Additionally, this study pinpointed crucial routes for technological compatibility, intention to use, and actual usage. According to this approach, technology compatibility somewhat mediates the distance between intention to use and actual technology use. Numerous studies have demonstrated that the higher the desire to use, the higher the perceived degree of compatibility with technology, and, eventually, the higher the actual usage is, the more intention there is to use [16,61,69,73,76,77,78,79]; their capacity to adjust in their careers increased as a result.

This research also revealed crucial links between Romanian preschool instructors’ desire to utilize technology, perception of enjoyment, compatibility with technology, and actual usage of technology. According to the model, the connection between preschool instructors’ intention to utilize technology and their actual usage of it was mediated by the relationship between compatibility and perceived enjoyment. Numerous studies have demonstrated that technology use increases with increased levels of intention to use [16,80] and that perceived enjoyment were positively associated with compatibility with technology perceptions [81,82]. Preschool instructors’ actual use is inversely correlated with their desire to utilize. Additionally, those with higher levels of perceived enjoyment would be more technologically compatible, leading to higher rates of real technology use. This model demonstrated how enhancements in reported enjoyment were connected to perceptions of compatibility with technology.

When preschool teachers had higher intention to use, they perceived the technology interaction as more enjoyable, had more compatibility perceptions with technology, which increased the level of actual technology use in class, and, in turn, improved individual career adaptability.

The cornerstone of lifelong learning is early childhood education. Additionally, a reliable and competent teaching staff is a crucial assurance for the long-term growth of early childhood education and care. Moreover, there is a serious lack of teachers in the current workforce, and more qualified instructors are urgently needed. As for a faster SDG 4 2030 Agenda adoption, supporting teachers in using inclusive and flexible teaching strategies, such as the use of technology in teaching at early ages, maximizes, in turn, the participation and learning of all children.

Although the state has recently placed great emphasis on the qualitative standards of early childhood education and has promoted a series of significant policies to support it, kindergarten teachers still struggle with issues such as low pay, intense work demands, and a lack of corresponding institutional guarantees.

Early childhood educators must immediately advance their professions and take the initiative to change with the times as they face unclear employment problems in the future. This lack of clarity is because work stability and professional growth can only be accomplished with strong career flexibility. In addition, our study indicated that preschool teachers who intended to employ technology in the classroom reported greater levels of perceived enjoyment, compatibility with technology, and career flexibility. Therefore, educational managers might consider offering courses to enhance perceived enjoyment and compatibility with technology for the training of preschool teachers.

There is still a dearth of research on how well technology is accepted in the field of preschool education despite the increased interest in using technology in educational settings, particularly in light of the COVID-19 crisis. Our findings may aid both theoretical and practical studies of technological acceptability. Theoretically, by evaluating the adaptable TAM’s relevance for preschool instructors during a public health emergency, our study adds to the body of empirical research that already exists. The TAM model has been shown to be resilient and applicable in both general and unique circumstances by both previous studies and our current work. To better explain the results of the Eastern and Western studies in the same discursive framework, our team has broadened the samples of technology acceptability study from Western teachers to Eastern European teachers, notably preschool teachers from nations such as Romania. Third, the partial mediation of perceived enjoyment and compatibility with technology over the relationship between intention to use and actual usage demonstrates the factors that may influence preschool teachers’ technological embrace.

Practically, our findings could offer advice to policy makers, preschool directors, and developers of educational technology systems. When designing a system, technology developers may take into account how to make instructional technology more user friendly [64]. Based on input from preschool instructors, technology companies might make educational tools easier to use by providing clear instructions or tutorials. The availability of technology specialists, learning communities, and core educational technology training could all be helpful support techniques for preschool managers in encouraging teachers to manage educational technology and, as a result, could improve the behavioral intentions of preschool instructors. To improve the technical skills of preschool teachers, education departments may think about developing programs for in-service teachers. The national professional standards for preschool teachers should be updated by the government, in particular the Ministry of Education, to include a requirement that they have the necessary technical expertise and educational skills.

## 5. Conclusions

The topic of young children with digital devices is being debated and discussed more and more. Teachers need assistance in comprehending how young children may use technology to create things rather than just consume them [83].

Research results indicate that early childhood educators’ opinions are changing; they are now more confident when using technology for business or personal purposes, but less confident when integrating technology with very young children. A good attitude towards the integration of technology is highly correlated with educators’ overall confidence and hours utilizing technology. Their use of technology with very young children and their pedagogy are related. Contrarily, training, or a lack thereof, has no real impact. It should also be emphasized how much more study is needed on how technology use affects very young children’s development [84].

This research employed a cross-sectional quantitative approach, allowing for the simultaneous investigation of several variables, such as, in our case, the intention to use, compatibility with technology, perceived enjoyment, and actual use of technology by preschool teachers. A valid TAM-ECEC scale was used to collect responses for the research variables. We further employed a correlational analysis to depict the relationship between variables. The next step was to test our hypothesis involving sequential mediating effects analyses, considering compatibility with technology and perceived enjoyment as complementary sequential mediators in the relationship between intention to use and actual use.

To further test the robustness of results, we have also modelled this relationship with a Radial Basis Function (RBF) neural network, a specific kind of Artificial Neural Network utilized for function approximation [61]. The RBF approach used the same hypothesized conceptual model, with the dependent variable being D5. actual use and the independent predictors being intention to use, compatibility with technology, and perceived enjoyment. This in-depth analysis has revealed the predictive significance of intention to use, compatibility with technology, and perceived enjoyment over actual technology use in class. This psychological mechanism that plays a role in between intention to use and actual technology use in preschool teachers adds to the topic literature, bringing more evidence on how to enhance preschool teachers’ technological acceptance behavior in class. It has been revealed that once the intention is set for embracing technology in early education, compatibility with technology and perceived enjoyment favorably impact actual technology use in class.

The main finding of this research is that compatibility with technology and perceived enjoyment complementary and sequentially mediate the relationship between preschool teachers’ intention to use and actual use. This study highlights one of the psychological mechanisms that influences the process from the intention to use to the actual use of technology in an ECEC educational setting. Similar results were found by Tao (2009) [85].

Similar findings also concluded that the subjective norm had the greatest impact on computer acceptance. Additionally, acceptability of computer technology was directly influenced by perceived utility and computer self-efficacy. The acceptability of computer technology, on the other hand, was indirectly influenced by perceived usability and individual creativity in educational technology [86].

There are certain limitations in this investigation. Being cross-sectional research, the findings are limited to showing correlations between factors rather than causality. The longitudinal research approach can be taken into account in the follow-up study. Second, because all the data in this study came from self-reporting questionnaires, it’s possible that participants’ replies were impacted by social norms. The follow-up study can use a variety of techniques, including peer and instructor evaluation. Third, this study has sampling restrictions because it only chose samples from 15 Romanian counties. Later research may collect responses from the entire nation or internationally. Lastly, this investigation did not fully investigate how certain possible mediator factors, such family socioeconomic position, parental employment, and other characteristics, affected how preschool instructors actually used technology. The parenting style [87,88,89,90,91,92,93,94] can further add valuable information. Only the probable influence mechanism on the four key variables was explored. The mediating impact of perceived satisfaction and compatibility with technology can be further explored in future research.

Given the aforementioned limitations, this research is the first to explore the connection between preschool instructors’ intention to utilize, reported enjoyment, compatibility with technology, and real technology usage. We looked at the link between the intention to utilize technology and the actual usage of it, as well as the mediating effects of perceived enjoyment and compatibility in succession. The results confirmed our prediction, specifically, that compatibility and perceived enjoyment are sequential mediators between intention to use and actual use. At the same time, intention to use indirectly predicted actual technology use level of preschool teachers through the mediating role of perceived enjoyment, compatibility with technology, and the sequential mediating role between compatibility and perceived enjoyment.

Despite the limitations highlighted above, this research is the first to use an RBF neural network technique to investigate preschool instructors’ desire to adopt technology and its affecting factors throughout the pandemic. Even if the TAM is more common in scientific literature, neural network RBF prediction techniques have not been the focus of prior investigations. This study helps identify potential factors that may affect preschool instructors’ intentions to use educational technology. The research findings may be used to create focused, effective strategies to increase the technological acceptance of preschool instructors and raise the standard of online learning in light of the SDG 4 2030 Agenda.

## Figures and Tables

**Table 1:** Correlation matrix.

		D3	D4	D5	D6
D3. Perceived enjoyment	Pearson’s r	—			
	*p*-value	—			
D4. Intention to use	Pearson’s r	0.822 ***	—		
	*p*-value	<0.001	—		
D5. Actual use	Pearson’s r	0.738 ***	0.795 ***	—	
	*p*-value	<0.001	<0.001	—	
D6. Compatibility	Pearson’s r	0.737 ***	0.696 ***	0.671 ***	—
	*p*-value	<0.001	<0.001	<0.001	—

Note. * *p* < 0.05, ** *p* < 0.01, *** *p* < 0.001.

**Table 2:** RBF Correct classification rates on data points.

Sample	Observed	Predicted					
		1.00	2.00	3.00	4.00	5.00	Percent Correct
Training	1.00	10	4	2	0	0	62.5%
	2.00	3	16	12	1	0	50.0%
	3.00	0	7	19	12	1	48.7%
	4.00	0	1	3	23	3	76.7%
	5.00	0	0	0	6	5	45.5%
	Overall Percent	10.2%	21.9%	28.1%	32.8%	7.0%	57.0%
Testing	1.00	3	3	0	0	0	50.0%
	2.00	4	5	2	1	0	41.7%
	3.00	0	0	15	5	0	75.0%
	4.00	0	0	1	7	1	77.8%
	5.00	0	0	1	1	5	71.4%
	Overall Percent	13.0%	14.8%	35.2%	25.9%	11.1%	64.8%

## Data Availability

The authors will make the raw data supporting the conclusion of this study available without restriction.
